# Prevalence and Risk Factors for Tobacco Use Among Tunisian Nursing Students

**DOI:** 10.1192/j.eurpsy.2026.10903

**Published:** 2026-07-31

**Authors:** A. Sdiri, I. Betbout, D. Charfi, Z. Ben Slimene, N. Faouel, M. Ben Mbarek, A. Ben Haouala, B. Amamou, A. Mhalla

**Affiliations:** 1 Psychiatry Research Laboratory LR05ES10 (Vulnerability To Psychoses), Faculty Of Medicine Of Monastir, University Of Monastir; 2 Psychiatry, Fattouma Bourguiba university hospital, Monastir; 3 UPSAT, Sousse, Tunisia

## Abstract

**Introduction:**

The use of psychoactive substances including tabacco among young people represents a growing public health concern. This population is particularly vulnerable due to academic pressure, social transition and increased autonomy, which may contribute to risky consumption behaviors. Understanding the prevalence and contributing risk factors is crucial for developing targeted prevention and support strategies among this population.

**Objectives:**

To assess the prevalence and risk factors associated with tobacco use among nursing students at a private institute in Tunisia.

**Methods:**

A cross-sectional analytical study was conducted involving 300 nursing students at a private higher education institute in Tunisia. Data were collected using a questionnaire, and tobacco dependence was assessed using Fagerström Test.

**Results:**

The study included 300 nursing students, predominantly female (63%), with most participants aged 20–21 years (49%). All respondents were single, and the majority (96%) had not repeated an academic year. Over half (54.3%) lived with their families. Overall, 33.7% of students reported having used addictive substances, with tobacco being the most commonly used (31.7%). Among tobacco users, 55.7% showed signs of probable dependence, while 12.6% reported occasional use. The average consumption was 5.05 cigarettes per day. Univariate analysis identified significant associations with tobacco use. Age was positively correlated with tobacco consumption (r=0.413, p<0.001). Male students were significantly more likely to smoke than females (25.7% vs. 6%, p<0.001). Academic level was not significantly associated (p=0.516), though a slight decrease in prevalence was observed across academic years. Notably, all students who had repeated a year reported smoking (p<0.001). Place of residence was not significantly associated with tobacco use (p=0.264). Multivariate analysis confirmed strong associations between tobacco use, age (OR ranged from 2.8 to 3.0), male sex, and year repetition, while first-year students were less likely to smoke (OR=0.48). These findings highlight age, sex, and academic status as key factors associated with tobacco use in this population.

**Conclusions:**

Tobacco use is prevalent among nursing students, with a concerning proportion showing signs of probable dependence. Age, male gender, and academic repetition emerged as significant risk factors. These findings highlight the need for targeted prevention strategies within university settings to reduce the initiation and progression of tobacco use in this vulnerable population.

**Disclosure of Interest:**

None Declared

